# Glyphosate Pollution Treatment and Microbial Degradation Alternatives, a Review

**DOI:** 10.3390/microorganisms9112322

**Published:** 2021-11-10

**Authors:** María Luisa Castrejón-Godínez, Efraín Tovar-Sánchez, Leticia Valencia-Cuevas, Marcos Eduardo Rosas-Ramírez, Alexis Rodríguez, Patricia Mussali-Galante

**Affiliations:** 1 Facultad de Ciencias Biológicas, Universidad Autónoma del Estado de Morelos, Cuernavaca 62209, Mexico; mlcastrejon@uaem.mx; 2 Centro de Investigación en Biodiversidad y Conservación, Universidad Autónoma del Estado de Morelos, Cuernavaca 62210, Mexico; efrain_tovar@uaem.mx (E.T.-S.); leticia.valencia@uaem.mx (L.V.-C.); 3 Centro de Investigación en Biotecnología, Universidad Autónoma del Estado de Morelos, Cuernavaca 62210, Mexico; marcos.rosas@uaem.edu.mx

**Keywords:** glyphosate, environmental pollution, microorganisms, pesticide, remediation

## Abstract

Glyphosate is a broad-spectrum herbicide extensively used worldwide to eliminate weeds in agricultural areas. Since its market introduction in the 70’s, the levels of glyphosate agricultural use have increased, mainly due to the introduction of glyphosate-resistant transgenic crops in the 90’s. Glyphosate presence in the environment causes pollution, and recent findings have proposed that glyphosate exposure causes adverse effects in different organisms, including humans. In 2015, glyphosate was classified as a probable carcinogen chemical, and several other human health effects have been documented since. Environmental pollution and human health threats derived from glyphosate intensive use require the development of alternatives for its elimination and proper treatment. Bioremediation has been proposed as a suitable alternative for the treatment of glyphosate-related pollution, and several microorganisms have great potential for the biodegradation of this herbicide. The present review highlights the environmental and human health impacts related to glyphosate pollution, the proposed alternatives for its elimination through physicochemical and biological approaches, and recent studies related to glyphosate biodegradation by bacteria and fungi are also reviewed. Microbial remediation strategies have great potential for glyphosate elimination, however, additional studies are needed to characterize the mechanisms employed by the microorganisms to counteract the adverse effects generated by the glyphosate exposure.

## 1. Introduction

**Figure 1:**
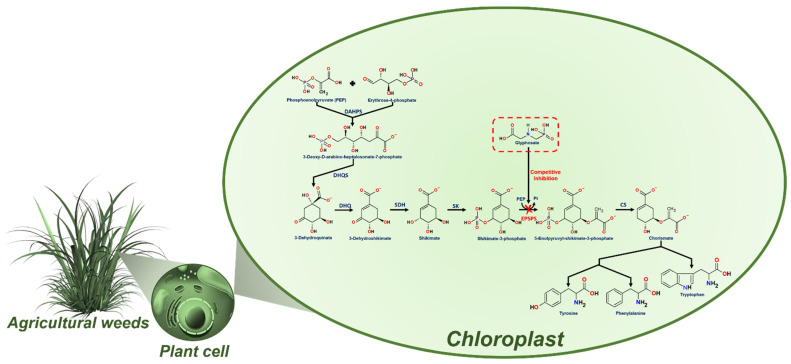
Glyphosate action mechanism through the inhibition of the shikimate pathway. Enzymes implicated in the pathway, DAHPS: 3-Deoxy-D-arabino-heptulosonate-7-phosphate synthase; DHQS: 3-Dehydroquinate synthase; DHQ: 3-Dehydroshikimate dehydratase; SDH: shikimate-5-dehydrogenase; SK: shikimate kinase; EPSPS: 5-Enolpyruvyl shikimate 3-phosphate synthase; and CS: chorismate synthase.

Glyphosate (*N*-Phosphonomethyl-glycine) is a broad-spectrum herbicide extensively used worldwide to eliminate weeds in agricultural areas, control vegetation in urban areas, and accelerate the harvest of several crops [1]. This phytotoxic activity of glyphosate was discovered at the beginning of the 70’s and it has been employed in different herbicide formulations since [2,3,4]. Glyphosate was patented as a pesticide with disrupting activity and lethal effects on a broad spectrum of plants with active photosynthesis [5,6]. Glyphosate herbicide activity has been attributed to its blocking effects on the shikimic acid pathway, through the inactivation of the key enzyme 5-enolpyruvylshikimate-3-phosphate synthase (EPSPS), due to its profile as a phosphoenolpyruvate analog (Figure 1). The blocking of the EPSPS enzyme prevents aromatic amino acids (phenylalanine, tyrosine, and tryptophan) biosynthesis and subsequent protein production [7], killing plants in a time of 1–3 weeks [8,9]. Glyphosate was proposed as a plant selective pesticide due to the absence of the shikimic acid pathway in animals and humans.

Since the glyphosate introduction to the market, it has been perceived as a less toxic weed control alternative, safe for agricultural workers and non-target organisms. In addition, glyphosate shows high effectiveness for eradicating weeds from crop fields before planting begins and for vegetation control on non-cultured areas, as the edge of roads and the sides of railways [10]. This perception of safety led to the growing popularity in the use of glyphosate-based herbicides among farmers worldwide [5], to the existence in the market of more than 750 products [11], and to be considered the most effective herbicide in all of history [12].

The worldwide use of glyphosate has become an environmental problem. The volume of application of this herbicide has been increasingly high in response to the appearance of resistant weeds, the introduction of transgenic glyphosate-resistant crops, and the adoption of new patterns in its agricultural uses, as its application as a desiccant to accelerate the harvest of grains and other crops [10,13]. Due to the intensive glyphosate use, the environmental presence and adverse effects of this herbicide are each time more evident. In addition, the toxic effects of the glyphosate-based commercial formulas are more significant due to the addition of adjuvants (surfactants), which have their own toxicity, but which also enhance the toxicity of glyphosate [8,14], highlighting the use of mixtures of polyethoxylated amines (POEA) [15]. Some POEA show toxicities 10–60 times higher than glyphosate in aquatic organisms and 4–50 times higher in mammals [16,17].

Glyphosate is known as a chelating agent that binds macro- and micronutrients, essential for several plant processes and the resistance to pathogens, sequestering of such nutrient can compromise plant resistance development, but also affect animals and human health [18]. The global increase in the intensive use of glyphosate-based herbicides causes the release and presence of residues of this chemical in the soil, water, and air, compromising the integrity of the surrounding environments and constituting a threat for the organisms that inhabit these polluted areas [19]. In this sense, different studies have evidenced the presence of glyphosate residues in surface soil, and their transport to the deep soil layers or to bodies of water near agricultural fields [20,21,22].

It has been documented that glyphosate is a highly water-soluble molecule. Hence, glyphosate can be dispersed through runoff processes to different superficial water bodies as rivers and lagoons or be absorbed by the soil particles [23] causing pollution and favoring its availability for the organisms that feed through sediment filtration [24]. For example, glyphosate is highly toxic for aquatic organisms such as algae, ferns, and fishes, while moderately toxic to amphibians, crustaceans, and earthworms [25]. Furthermore, in recent studies, glyphosate residues have been identified in several agricultural products consumed by human populations [2,26]. Moreover, residues of this agrochemical were detected in food, human blood and urine, and water supplies. In a contrasting way, the initial reports considered glyphosate as a safe molecule with low toxicity on non-target organisms [2]. However, recently, the International Agency for Research on Cancer (IARC) classified glyphosate as a probable carcinogen [11], which can negatively affect mammalian biology through multiple ways, like those related to genotoxic effects and its ability to trigger oxidative stress [2].

The environmental and toxicological adverse impacts of glyphosate have been broadly reviewed. Several studies related to the identification of glyphosate in the environment and farm products destined for animal and human consumption have been published. Moreover, different toxicological studies have evaluated the toxicity of glyphosate on different organisms and the adverse effects of both occupational and non-occupational exposure to this herbicide in humans. However, reviews covering the treatment alternatives and the potential of microorganisms for glyphosate biodegradation are still limited. Since glyphosate-based herbicides are the most widely used globally, they promote severe effects on the environment and human health. This review aims to describe its environmental impacts, the risk for environmental and human health, the analytical methodologies developed for the detection and analysis of contamination by this type of agrochemicals, analyze resent research in microbial glyphosate degradation, and delves into the biodegradation mechanisms employed by microorganisms. Finally, we propose strategies for its control and prevention as well as some alternatives for bioremediation of polluted environments by glyphosate.

## 2. Environmental Impacts of Glyphosate

For 40 years, glyphosate has been widely used as an active chemical component of more than 750 commercial herbicides under the assumption that its side effects were minimal [11,27]. However, this compound’s intensive and large-scale use in industrialized and developing countries motivated the scientific community to evaluate the risks associated with the possible accumulation of its residues in various environmental systems and its effects on environmental and human health [28]. In this sense, recent evidence shows that herbicides containing glyphosate can contaminate the soils around the treated areas, glyphosate is adsorbed to clays and organic matter, slowing down its degradation by the action of microorganisms, leading to an accumulation in soils over time [29,30,31], the persistence of glyphosate in high clay content soil reaches more than a year [31,32].

Glyphosate has a great capacity for adsorption in the clay and organic matter present in soils, being considered as a low mobility compound, so that it is assumed that it does not represent a significant risk for the contamination of the water sources [33,34,35]. However, the scientific literature has reported the presence of this compound dissolved in groundwater [36,37], the transport of soil particles with glyphosate in surface water [21,37,38], as well as its adsorption in the sediments of water bodies [35]. Moreover, the glyphosate deposited in the first centimeters of the superficial soil layer is susceptible to wind erosion and atmospheric transport [39,40], consequently, glyphosate has been detected in the air, rain, and water from melting snow [21,41]. Finally, glyphosate has also been reported in seawater, where it is very persistent [42] and in drinking water [43].

Resulting from its accumulation and persistence in the soil, glyphosate can affect exposed organisms in this environmental compartment [44]. For example, it has been reported that glyphosate can affect the activity of soil microorganisms that are involved in biogeochemical cycles, the mineralization of organic remains, the immobilization and solubilization of minerals, and the degradation of other xenobiotics [45,46,47]. Likewise, a reduction in the reproduction rate, biomass, and DNA damage in earthworms has been reported [48,49,50], as well as adverse effects in other small size organisms, such as nematodes, distributed in soils [51]. Moreover, in plant species, the direct effects of their exposure to glyphosate are related to the inhibition of the activity of antioxidant enzymes and the induction of reactive oxygen species (ROS), which promote cell damage and physiological alterations in processes such as photosynthesis and the production of secondary metabolites [52]. Furthermore, traces of this compound can be detected in plant tissues of temperate zone species up to more than 12 years after the treatment [4]. Glyphosate indirectly changes the rhizosphere microbiome, which affects plant health [28,53].

In water bodies, the negative impacts of glyphosate have been observed in organisms such as protozoa, mussels, crustaceans, frogs, and fish [28]. Similar to that reported in terrestrial ecosystems, the presence of glyphosate in fish produces metabolism alterations, leading to the overproduction of reactive oxygen species and oxidative stress resulting in kidney damage [54]. Likewise, other studies have linked glyphosate exposure to DNA damage and chromosomal alterations in fish [55,56]. The presence of glyphosate not only has effects at the level of individual organisms, but alterations in the interactions between species have also been documented. As an example, an increase in the levels of susceptibility of fish to its parasites has been documented [57,58]. Similarly, unwanted effects of glyphosate exposure have been reported in bee species that provide valuable ecosystem services such as pollination [59]. Finally, glyphosate has been detected in animal feed, animal meat, and urine, as well as in the food intended for human consumption, which is why the presence of this herbicide has been detected in samples of breast milk and urine [5]. Another additional environmental risk associated with the presence of glyphosate, which has not been adequately considered, is that it is a potent mineral chelator [18] whose application can lead to the reduction of macro and micronutrients that are essential cofactors in many biological processes of glyphosate-treated plants and potentially also for the organisms that feed on them. Consequently, a reduced supply of nutrients in the treated plants can compromise their resistance to diseases. In the case of humans and other animals that consume food obtained from plants treated with glyphosate, the residues of this herbicide and the reduced levels of nutrients can also have an impact on their health [18,60]. Therefore, to minimize its environmental and human health impacts, monitoring and detection of its presence in different environments, as well as the evaluation of exposure to this herbicide in humans, is of utmost importance.

## 3. Glyphosate Human Health Threats

Humans have been exposed to glyphosate directly through occupational exposure or indirectly through various sources [61]. Occupational exposure includes agricultural workers, farmers, gardeners, and people who work in plants that process glyphosate [62]. These people can be exposed to glyphosate through inhalation, dermal and ocular contact. In contrast, the indirect exposure includes the consumption of water or food contaminated with glyphosate residues [63] or the environmental exposure to residues or products of its transformation, such as aminomethyl phosphonic acid (AMPA) in environmental matrixes such as air, water, or soil [61,62].

The mode of action of glyphosate consists of the inhibition of the enzyme EPSPS involved in the biosynthesis of the aromatic amino acids tyrosine, tryptophan, and phenylalanine through the shikimate pathway in plants [64]. Therefore, glyphosate was proposed as a low toxicity compound for non-target organisms and was considered relatively safe for humans, according to the results of different exposure studies carried out in rodents, chickens, and amphibians [12,13,65,66].

More recently, it was determined that the toxicity of commercial herbicides based on glyphosate is exacerbated by the presence of surfactant compounds in the formulation, being polyoxyethyleneamine (POEA) the most common [5,61]. This compound uncouples elements of phosphorylation oxidative stress, causing oxidative stress and cardiotoxicity [67]. Thus, in 2015, the World Health Organization reclassified glyphosate as a possible human carcinogen [11,68,69]. Glyphosate reclassification into the group 2A of the International Agency for Research on Cancer (IARC) was based on the review of the accumulated evidence provided by experts in cancer and toxicology, which has contributed to a better understanding of the toxicity of the compound for all kinds of exposed organisms in natural areas, and in experimental animals, and its mechanisms of action [70].

Among the most relevant information on the effects caused by glyphosate exposure in humans are the studies by Samsel and Seneff (2013b) [71], which indicated that exposure to the herbicide represented the main factor causing gluten intolerance and gastrointestinal disorders, as well as interference in the assimilation of micronutrients such as iron, cobalt, molybdenum, copper, and amino acids such as tryptophan, tyrosine, methionine, and selenomethionine. Subsequently, Samsel and Seneff (2013a) [60] and Schinasi and Leon (2014) [72] evidenced an association between non-Hodgkin’s lymphoma and agrochemical exposure. In particular, the latest work showed that occupational exposure to glyphosate increases the relative risk of developing this disease and the development of B-cell lymphoma. Other reports of chronic exposure to the herbicide in human populations show the association with conditions such as allergies, and asthma [73], cardiovascular diseases [74], autism, and chronic degenerative diseases such as multiple myeloma [75]. Cytotoxic damage has also been reported in chorioplacental cells of humans, which triggers inhibition in the synthesis of progesterone as a secondary effect [76]. Recent reviews suggest that glyphosate and glyphosate-based herbicides promote cytotoxic and genotoxic effects, a significant increase in oxidative stress, disruption of the estrogen pathway, adverse effects on various cognitive processes, and an association with the development of certain cancers [77,78]. The studies mentioned above compile evidence of the high glyphosate toxicity and establish this compound as a menace to the health of the agricultural population that has a history of direct exposure and for whom the exposure has been indirect through the consumption of food or water with residues of glyphosate. Human individuals exposed to this compound have presented multiple organ toxicity, nephrotoxicity, hepatotoxicity, gastrointestinal, cardiovascular, and respiratory effects [62,79].

## 4. Environmental Risk Assessment through Glyphosate and Its Metabolites Detection

Several methodologies have been developed to detect the presence and amount of glyphosate in different environmental samples, including soils, sediments, plant material, surface water, and human fluids [80,81]. However, the chemical characteristics of glyphosate, such as its high solubility in water, insolubility in organic solvents, non-volatility, high sorption to the soil, as well as the lack of fluorophores and chromophores groups in its molecular structure make its detection and quantification in environmental samples difficult [81,82]. To improve the analytical detection of glyphosate and AMPA, the primary toxic metabolite derived from glyphosate degradation, it is necessary to carry out a first derivation step before the analytical determination [83]. The derivatizing agents used for the detection of glyphosate modify its properties and make it detectable by various spectroscopic techniques. A recent review revealed that derivatization is commonly done by acylating agents, alkylchloro or fluoro formates, benzenesulfonyl, and phthalaldehyde [34]. For the glyphosate and AMPA detection through HPLC analyses several aromatic reagents have been employed as derivatizing groups such as 2,5-dimethyl benzene sulfonyl chloride, *p*-toluenesulphonyl chloride, *o*-nitrobenzene-sulfonyl chloride, *o*-phthalaldehyde (OPA), 9-fuorenylmethylchoroformate (FMOC) [84,85,86,87]. In the same analytical technique, the derivatization of glyphosate and AMPA with OPA, after the chromatographic column separation, allows their detection and analysis through a fluorescence detector (FLD).

Alkyl chloroformates like isobutyl chloroformate and isopropyl chloroformate replace the active hydrogen atoms present in the glyphosate molecule with an aliphatic or aromatic group and convert them into their ester derivatives. These ester derivatives are less polar and more stable than glyphosate. These transformations improve the chromatographic properties of glyphosate, and hence its detection by gas chromatography becomes easier. There is another derivatizing reagent called FMOC, which transforms glyphosate into a carbamate derivative. These carbamate derivatives are more stable and show good chromatographic properties. The fourth type of derivatizing reagent is 4-methoxybenzene-sulfonyl fluoride which converts glyphosate into sulphonamide derivative, whereas OPA changes it into phosphoramide derivative. These sulfur and phosphorus derivatives bring fluorescent properties to glyphosate and make detection easy by ‘flame photometric detector’ through liquid chromatography. Another commonly used reagent, ninhydrin, converts glyphosate’s amino group into a highly fluorescent compound that can be detected easily by ultraviolet spectroscopy. The reagents like 4-chloro-7-nitrobenzofurazan, naphthalene2,3-carboxaldehyde, and fluorescein isothiocyanate also introduce fluorescence into glyphosate molecules [34].

Methods that have been used for glyphosate and AMPA extraction, derivation, pre-concentration, and quantification in environmental samples [88], include UV capillary zone electrophoresis (CZE) coupled to conductivity detection (CD) and UV detection [89], condensation nucleation light scattering detection (CNLSD) [90]; high performance liquid chromatography (HPLC) coupled to mass spectrometry (MS) [91]; ion chromatography (IC) with CD and inductively coupled plasma MS (ICPMS), capillary electrophoresis (CE) with capacity couple contactless conductivity detection (C4D) [91]; fluorescence ICP-MS [92]; electrochemical detection [93]; HPLC coupled with Tandem MS [94]; a flow injection (FI) system with electrochemiluminescence (ECL) detection [95], enzyme-linked immunosorbent assay (ELISA) [96,97,98,99]; gas chromatography (GC) coupled to MS [97], time-of-flight MS (TOF–MS) [100].

In general, most of these technologies require high-end equipment and resources, making glyphosate detection and quantification expensive and slow [101]. Undoubtedly, having efficient methodologies and techniques to detect the presence and quantity of glyphosate, its metabolites, or adjuvants are necessary to improve the knowledge of the potential risks to humans and environmental health from exposure to this herbicide, so this will be a field of great relevance in the coming years.

## 5. Need for Pollution Prevention and Treatment

The concentration of glyphosate in the herbicidal commercial formulations may vary between 0.94 and 94 *w*/*w*%, 36% being the most common proportion [102]. The selection of the commercial glyphosate formulation and the dosage applied to the crop fields are established according to the needs of farm producers. However, the recommended application dose of commercial formulation (36% pure glyphosate) is around 1.5–6 L (0.54–2.2 kg) by hectare (ha). In the European Union, glyphosate use in annual crops systems such as corn and wheat ranges from 0.5 to 2.7 kg/ha, while in perennial crops such as olive groves and vineyards, glyphosate dose ranges from 0.2 to 2.5 kg/ha [103].

After application, glyphosate is quickly attached to the soil, where it and AMPA may persist from a few days up to a year, while in water, due to its high solubility, glyphosate enters water bodies and its half-life can reach three months [104]. In a study carried out in soil of different countries of the European Union, glyphosate and AMPA residues were identified in concentrations from 0.5 mg/kg up to values higher than 1000 mg/kg, the AMPA concentrations were higher with respect to glyphosate in all sampled soils [40]. In several countries of Europe, North and South America, the reported glyphosate concentration ranges from 0.1 to 328 µg/L in surface water samples, while from 0.7 to 2.5 µg/L in groundwater samples [28].

Glyphosate shows different range toxicities according to the evaluated organisms, in acute toxicity studies glyphosate may cause mortality in aquatic organisms such as *Daphnia magna* (EC_50_ 40 mg/L), *Lepomis macrochirus* (EC_50_ 47 mg/L) and rainbow trout *Oncorhynchus mykiss* (EC_50_ 38 mg/L), while in human cells, cytotoxicity has been observed in concentrations higher than 10,000 mg/L. With respect to oral exposure, glyphosate toxicity has been observed in rats (90 days exposure) at concentrations of 300 mg/kg/day, while in dogs (90 days exposure) toxic effects were observed at 1000 mg/kg/day [17].

The toxicity of glyphosate has been evaluated in rodents, through oral exposure (technical glyphosate; >80–90% purity), we have observed that the studies using commercial formulations using animals, are limited. In animals, the toxic effects of glyphosate oral exposure vary according to the dosage, at 175 mg/kg/day in acute exposure (≤14 days) diarrhea is observed, concentrations ranging from 300–460 mg/kg/day may cause diarrhea, salivary glands cytoplasm changes, and inclusively death in intermediate exposure (15–364 days) and gastric mucosa inflammation and salivary glands cytoplasm changes in chronic exposure (>365 days); these adverse effects may be extrapolated to humans [102].

Although glyphosate as an active chemical component of herbicides may have low toxicity to exposed organisms, the effects of chronic exposure are not known [61]. Another important risk is that it has been shown that the presence of its metabolites (AMPA) and its adjuvants as POEA in commercial formulations may have higher levels of toxicity than glyphosate alone [101]. In addition, the persistence of glyphosate, adjuvants, and metabolites in the environment may be greater than previously considered, which, together with the difficulties in their detection [105], implies a greater risk for exposed organisms. In this sense, it has already been documented that glyphosate has caused toxic effects in non-target organisms found in soil and water, including various plants, animals, and bacteria [34,106]. Another serious concern is that glyphosate may compromise biodiversity as it has reduced the availability of weeds that serve as an important food source for many species [34]. This information makes it evident that our knowledge of glyphosate’s ecological safety, its behavior in natural environments, how it interacts with wild organisms, and its degradation routes are insufficient. In addition to those mentioned above, the association of glyphosate with carcinogenesis in humans and other chronic diseases reveals the urgent need to prevent contamination in natural environments (soil and water bodies) and the search for alternatives for its removal, control, and treatment in the environment.

## 6. Physicochemical Treatments for Glyphosate Remediation

Physicochemical processes such as adsorption, membrane filtration, and coagulation have proven to be efficient and economical for removing glyphosate [107,108]. Adsorption is a widely used process for treating and purifying water contaminated with glyphosate due to its simplicity, non-toxicity, low-cost design, and high efficiency. The adequate selection of adsorbent material is crucial, among the materials that have been used as adsorbents to remove glyphosate from wastewater are clay substances [109], activated carbon [110,111], zeolite [112]; biochar [113,114], graphene oxide and iron-based adsorbing materials [115], resins [116,117]. The glyphosate adsorption process occurs through physical and chemical interactions between the functional groups of the glyphosate molecule (-COOH, -NH_2_, and -PO(OH)_2_) and the surface of the adsorbent [107]. In general, it has been found that under acidic conditions, the adsorption of glyphosate by different adsorbents is more favorable, so it has been proposed that pH is one of the most determining factors affecting the glyphosate adsorption process [108,113]. Likewise, the concentration of the pollutant, the temperature, the adsorbent dose, and the ionic strength are also key factors for the overall efficiency of this process [115]. Although adsorption is an efficient method for the treatment of glyphosate in low concentrations, some drawbacks limit its application in practice: (1) successful glyphosate adsorption requires acidic conditions, it is not recommended to drastically change the pH of the wastewater that later could be released into the environment, so that the treated water needs a neutralization step prior to the its environment release; (2) there is no selectivity of adsorbents for glyphosate, which is a disadvantage considering that wastewater contains many other pollutants; (3) most of the studies have been at the laboratory level using ex-professo prepared glyphosate aqueous solutions; and (4) the removal of the residue after adsorption continues to be a problem that also needs to be considered [107,108]. Using this technology as the principal treatment is not recommended (although the affinity of glyphosate with some of the adsorbents is quite good), so it has been suggested to use it as a possible secondary treatment when the concentration of other pollutants is low.

Another technology that has been used to treat glyphosate is membrane filtration [118,119,120]. This system works as a barrier for matter transport of an influent stream and separates it into two effluent streams: the permeate and the retentate or concentrate [118]. An advantage of the membrane filtration process is that it does not break down the glyphosate into molecules so that it does not produce harmful by-products that could be more toxic than the original contaminant. In addition, the contaminant could be recovered after the membranes are saturated. However, a disadvantage of membrane filtration could be its specificity to reject specific pollutants since, in real conditions, the characteristics and composition of the water could change, compromising the efficiency of the filtration process [107]. Additionally, in several studies, glyphosate removal has not been done in real polluted water. Therefore, for successful membrane filtration, it is necessary to evaluate in more detail the effect of natural organic matter on the efficiency of this system for water contaminated with commercial glyphosate formulations.

Advanced oxidation processes include numerous techniques based on the formation of strong oxidants agents, such as hydroxyl radicals (OH^−^), which can help to degrade organic pollutants, conducting to its complete mineralization in CO_2_, H_2_O, and inorganic salts [121,122]. Among them, we can mention Fenton reagent, photo-Fenton, ozonation, photocatalysis, H_2_O_2_-UV, and electrochemical treatments [108,122]. For example, Fenton oxidation has been a successful technology for glyphosate treatment with the advantages of simple operation, no mass transfer limitation, and easy implementation as a standalone or hybrid system [123]. Through this process, the degradation of toxic organic molecules is achieved by employing strong chemical oxidizing species.

However, the continuous loss of oxidants and iron ions, the formation of solid sludge, and the high costs and risks related to the handling, transport, and storage of reagents are some drawbacks in the Fenton process [124]. To avoid the disadvantages that are associated with the Fenton process, combined processes have been proposed, such as the use of electro-Fenton and photo-Fenton treatments. Application of this alternative Fenton process seek to enhance the production of OH- radicals through electrochemical reactions or irradiation with ultraviolet or visible light, respectively [125]. Complete elimination of glyphosate and good mineralization have been reported through the use of electro-Fenton and photo-Fenton treatments [126].

However, another disadvantage is that this technology has not been tested in real systems [108]. Electrochemical oxidation is one of the cleanest technologies to degrade glyphosate compared to other advanced oxidation processes [107]. It offers high efficiency without adding chemical products [127,128]. This technology is based on anodic oxidation reactions to degrade organic matter to its complete mineralization or even less complex molecules that are biodegradable [129]. For this degradation process, pH, initial glyphosate concentration, electronic composition, electrolysis, and current density are important parameters [130,131]. The use of electrochemical oxidation has been efficient for the degradation of glyphosate since it reaches almost the total mineralization of this pollutant [127,131,132]. However, the main disadvantages of the process are associated with the high cost of the electrodes, electrode fouling, corrosion, and the possible formation of environmentally toxic intermediates [133].

Oxidation by ozonation can also effectively treat wastewater containing low concentrations of glyphosate in the shortest possible time. In this technology, two mechanisms of glyphosate oxidation are used: direct oxidation by ozone (O_3_) or indirect oxidation by hydroxyl radicals [108]. Through this process, complete degradation of glyphosate by ozonation has also been achieved [134]. In addition, high glyphosate and AMPA removal efficiencies have been reported when using O_3_ and H_2_O_2_ simultaneously, in a short reaction time [135]. However, its application in practice is complicated because O_3_ is unstable under normal conditions, has low solubility in water, is expensive, and the mass transfer of O_3_ limits its performance [108].

Recently, the application of combined technologies has attracted the scientific community’s attention, who are looking for more efficient alternatives for the degradation of glyphosate. For example, Xing et al. (2018) [136] reported 100% removal of glyphosate in wastewater samples through the combined use of electrochemical oxidation and adsorption. Another option has been the combination of biological treatment and physicochemical processes for the treatment of water for drinking and rainwater with glyphosate residues. This alternative has been successfully employed in the treatment of polluted soils through biofilters that use plants [137] and microorganisms [138], with glyphosate removal efficiencies of 99 and 90%, respectively. These combined processes represent interesting alternatives that could favor cost and efficiency in glyphosate degradation and therefore, further studies are required. As was highlighted, glyphosate-contaminated wastewater or soils could be efficiently treated with the technologies mentioned above, as they can degrade glyphosate and other pollutant molecules. However, to date, most of the tests have been at the laboratory level, so more studies are necessary to allow their implementation in real scenarios.

Another appropriate solution seems to be the use of bioremediation alternatives in which the use of microorganisms capable of degrading glyphosate into biologically safer compounds are employed to reduce environmental exposure risks.

## 7. Glyphosate Biodegradation Alternatives

Until recently, intensive use of the herbicide glyphosate was not considered an environmental risk, so research in the field of its bioremediation was limited [27]. However, the presence of glyphosate at different environmental matrixes has been related to human and ecosystem threats [5,107,122]. The environmental risks associated with glyphosate have driven the research and development of effective and environmentally friendly strategies for the cleaning and restoration of contaminated areas with the presence of this herbicide. In general, several methodologies, such as biological and physical methods, as well as advanced oxidation processes, have been successfully employed for glyphosate elimination [107,108,139].

Different authors have reported the degradation of glyphosate through different microorganisms such as bacteria, actinomycetes, and fungi; however, bacteria are the most reported microorganisms [102,108,140,141]. The biodegradation of glyphosate through the use of microorganisms has been considered a safe, low-cost, and reliable alternative to removing this xenobiotic from water and soil [140]. In this case, glyphosate degradation occurs because species of bacteria and fungi can use this compound as a source of nitrogen, carbon, and phosphorus, transforming it into new compounds through different degradation pathways [142,143]. Among the microorganisms (bacteria and fungi) used for the biological degradation of glyphosate are *Achromobacter* spp., *Agrobacterium radiobacter*, *Alcaligenes* sp. GL, *Arthrobacter* spp., *Bacillus cereus* CB4, *Ochrobactrum* spp., *Pseudomonas* spp., *Aspergillus niger*, *Aspergillus oryzae* A-F02, *Penicillium chrysogenum*, *Trichoderma harzianum*, among others [107]. The most common bacteria for glyphosate biodegradation are *Pseudomonas* spp. [144], which use glyphosate as a source of phosphorus [108]. It was recently documented that the employment of co-cultures of *Pseudomonas* sp. and *Bacillus* sp. is an effective strategy to degrade glyphosate in contaminated soils and aquifers since these bacteria can use glyphosate as the sole source of phosphorus [143]. Some disadvantages of biological treatments are that they cannot achieve high efficiency in the mineralization process due to the generation of by-products such as AMPA [141] or sarcosine [145], they require long periods of adequate growth time and conditions to achieve the highest degradation efficiencies [107,141]. In addition, for a more effective removal process, it is preferable for bacteria to use glyphosate as a source of carbon and not phosphorus since they have a greater demand for the former for their growth and metabolism [108]. Due to the generation of by-products, combining biological treatments with other processes can improve the efficiency of degradation of glyphosate and its metabolites.

### 7.1. Bacterial Degradation of Glyphosate

**Figure 2:**
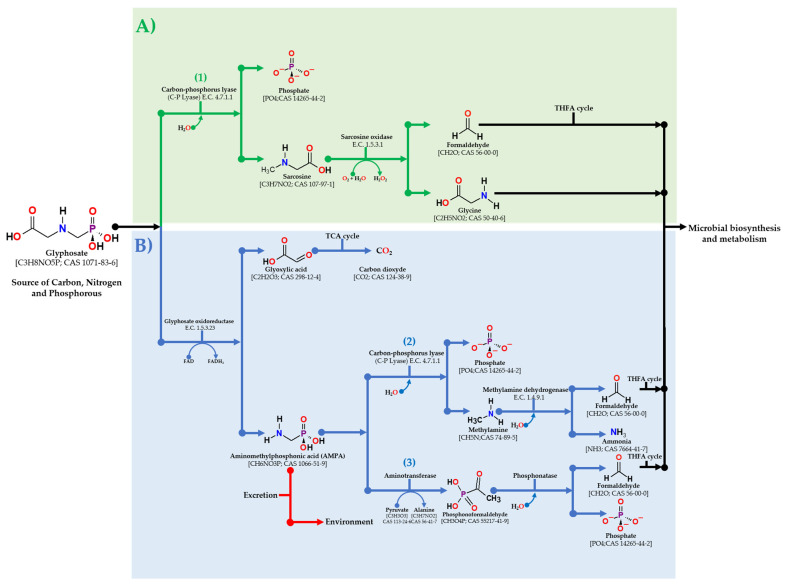
Principal glyphosate biodegradation pathways in bacteria. (**A**) Carbon-phosphorous lyase pathway (C-P lyase), (1) glyphosate degradation through sarcosine release as reported in *Pseudomonas* sp. PG2982 [148]. (**B**) Glyphosate oxidoreductase pathway (GOX pathway), (2) AMPA degradation in methylamine as reported in *Arthrobacter* sp. GLP-1 [149] and *Flavobacterium* sp. GD1 [150], and (3) alternative AMPA degradation pathway through the aminotransferase activity as reported in *Ochrobactrum anthropi* [151].

Different bacteria can metabolize glyphosate; its biodegradation generates the formation of metabolites that are used as a source of carbon, nitrogen, and phosphorus, elements that are essential for its development [146]. Bacteria can degrade glyphosate through two metabolic pathways. In the first pathway, the activity of the enzyme glyphosate oxidoreductase (GOX) breaks the glyphosate molecule in two derived metabolites, the glyoxylate, which enters the tricarboxylic acid cycle and, as a result of its complete oxidation, generates carbon dioxide, and on the other hand, aminomethylphosphonic acid (AMPA), which with the help of the enzyme carbon-phosphorous lyase (C-P lyase) is hydrolyzed into phosphate and methylamine. The latter metabolite is transformed into ammonia (direct source of nitrogen) and formaldehyde that enters into the tetrahydrofolate (THFA) cycle. In the same pathway, through alternative reactions, AMPA is transformed with the help of the enzyme aminotransferase to phosphonoformaldehyde, which in turn is transformed by the enzyme phosphonatase into phosphate and formaldehyde which is also entered into the THFA pathway. The second pathway reported for the biodegradation of glyphosate involves the enzyme C-P lyase, which, through its hydrolytic activity, generates phosphate and sarcosine; in a subsequent step through the activity of the enzyme sarcosine oxidase, sarcosine is transformed into the amino acid glycine, which is used directly for metabolism and microbial biosynthesis, and formaldehyde that is entered into the THFA cycle [141]. To better understand the glyphosate biodegradation process, the interaction of glyphosate and AMPA with the key biodegradation enzymes GOX, and C-P lyase was studied through molecular docking and molecular dynamics. The results of these in silico experiments reveal the establishment of stable interactions of these metabolites with the active site of GOX and C-P lyase. Hydrogen bonds, van der Waals, and hydrophobic were prosed as the most important interactions [147]. In Figure 2, the main glyphosate biodegradation pathways in bacteria are shown.

Table 1 lists recent studies related to bacterial-mediated degradation of glyphosate. In general, 28 species of bacteria were documented belonging to 13 genera with the capacity for glyphosate degradation. Their importance by the number of studies addressing them, shows the following pattern: *Bacillus* > *Ochrobactrum* > *Agrobacterium* = *Achromobacter* = *Burkholderia* = *Ensifer* = *Pseudomonas* = *Rhizobium* > *Acidovorax* = *Comamonas* = *Lysinibacillus* = *Sinorhizobium* = *Streptomycete.* Overall, the herbicide degradation efficiency among the bacterial strains listed in Table 1, ranged from discrete percentages (37%) to its complete degradation (100%). Additionally, when data are organized into categories according to the bacterial degradation efficiency of glyphosate: low (37–58%), intermediate (57–78%), and high (79–100%). According to the information shown in Table 1, the percentage of bacterial species belonging to each degradation category followed the next pattern: low (39%) > high (36%) > intermediate (25%). The genera with lowest degradation efficiency for glyphosate were *Ochrobactrum*, *Agrobacterium*, *Achromobacter*, *Bacillus*, *Pseudomonas*, *Rhizobium*, *Sinorhizobium*, and *Ensifer*, and with intermediate degradation efficiency were *Bacillus*, *Ochrobactrum*, *Pseudomonas*, *Bacillus*, *Burkholderia*, and *Achromobacter*, and finally, the genera with the highest degradation efficiency were *Lysinibacillus*, *Bacillus*, *Rhizobium*, *Streptomyces*, *Burkholderia*, *Comamonas*, and *Ochrobactrum*. Only *Pseudomonas aeruginosa* and *Bacillus cereus* were classified into two levels for glyphosate degradation, intermediate-low and intermediate-high, respectively (Table 1). The fact that these two species were assigned to more than one glyphosate degradation category may be because both species were exposed to a wide range of glyphosate concentrations. Bacterial strains can degrade glyphosate through either the glyphosate oxidoreductase (GOX) pathway, the carbon-phosphorus lyase (C-P lyase) pathway, or both (Table 1).

### 7.2. Fungal Degradation of Glyphosate

A limited number of fungal genera such as *Aspergillus*, *Fusarium*, *Penicillium*, and *Trichoderma* have been reported with the ability to degrade the herbicide glyphosate and use it as a source of carbon, phosphorus, or nitrogen [142,166,168,169]. Kulikova et al. (2020) [169] report that the biodegradation of the herbicide can be carried out both with the formation of AMPA and sarcosine, the latter being the main degradation pathway. For their part, in the study of Njoku et al. (2020) [164] fungal strains were identified capable of degrading glyphosate; this study points out that the selected fungi biodegraded the herbicide through GOX pathway due to the presence of the AMPA metabolite, in exception of *Aspergillus fumigatus* FJAT-31052. Despite these studies, the metabolic processes related to the degradation of glyphosate in fungi have not been fully described. Therefore, it is necessary to continue researching to identify the enzymes involved and to evaluate the effects of the herbicide on these organisms [142]. The studies by Mesnage et al. (2020) and Guo et al. (2021) [170,171] are examples of some approaches to understanding the effects of glyphosate on *Aspergillus nidulans* and *Fusarium verticillioides*, respectively, through transcriptomic approaches. Table 1 presents recent studies related to the fungal-mediated degradation of glyphosate. In general, 13 species of fungi were documented belonging to four genera with the capacity for glyphosate degradation; their importance by the number of studies addressing them, showed the following pattern: *Aspergillus* > *Trichoderma* = *Penicillium* > *Trichoderma*. Overall, the herbicide degradation efficiency among the fungi strains listed in Table 1, ranged from 8% to 87% in *Trichoderma* sp. and *Aspergillus niger* MT871999, respectively. Additionally, when data are organized into categories according to the fungi degradation efficiency of glyphosate, low (8–35%), intermediate (36–63%), and high (64–90%), we detected that the percentage of fungi species belonging to each degradation category shows the following pattern: high (62%) > low (31%) > intermediate (15%). The species with lowest degradation efficiency for glyphosate were *Trichoderma* sp., *Aspergillus flavus* EFB01, and *Aspergillus* 2B112, those with intermediate degradation efficiency were *Aspergillus oryzae* AM1 and *Penicillium* 4A21, and finally, the species with the highest degradation efficiency were *Aspergillus oryzae* A-F02, *Trichoderma harzianum* MT871998, *Trichoderma gamsii* P2-18, *Aspergillus fumigatus* FJAT-31052, *Penicillium simplicissimum* SNB-VECD11G, *Aspergillus niger* APBSDSF96, *Aspergillus flavus* JN-YG-3-5, and *Aspergillus niger* MT871999.

### 7.3. Algae Degradation of Glyphosate

Only the algae species *Oscillatoria limnetica* has been documented with the ability to degrade the glyphosate. Overall, the herbicide degradation efficiency ranged from 38% to 85%, the degradation variation percentages of this particular species may be a consequence of its exposure to different glyphosate concentrations (Table 1). It is important to note that the low number of studies on degradation and bioremediation of glyphosate using algae, offers a window of opportunity to increase the number of analyses using algae species. Algae species may be a good study model for bioremediation strategies due to (i) its sensitivity to pollutants, (ii) they are considered cost-effective, (iii) rapid cell cycle (iv) tests can reach acute exposure in a few hours, (v) sublethal responses of chronic toxicity can be assessed within a very short period (days), (vi) algae responses are at population level and not just at individual cells, which makes it possible to infer the responses of subsequent generations. In addition, the bioremediation of environments impacted by glyphosate that promote a detrimental impact in all levels of biological organization has been identified as one of the pending challenges in environmental toxicology, therefore, we require to find new study models such as algae species with high potential for herbicide degradation that help to return to a healthy environment along with other bioremediation techniques.

According to the information shown in Table 1, bacteria is the most studied group of microorganisms for glyphosate biodegradation, with 14 reports, followed by fungi with 5 reports, and finally algae with just 1 report. The glyphosate maximum biodegradation efficiencies in bacteria ranged between 90 and 100%, while in fungi and algae the maximum biodegradation efficiencies reached values below 90%. Moreover, higher glyphosate concentrations have been evaluated in studies carried out in bacteria (5–14,400 mg/L), in comparison to fungi (100–1690 mg/L) and algae (5–20 mg/L). According to these reports, bacteria seem the group of microorganisms with the higher potential for the implementation of glyphosate biodegradation strategies.

## 8. Conclusions and Future Perspectives

Glyphosate is the herbicide most employed worldwide. Its mechanism of action over weeds is related to the inactivation of the enzyme EPSPS in the shikimate pathway and the subsequent inhibition of aromatic amino acids biosynthesis. The absence of this metabolic pathway in animals and humans led to the general belief of the safeness of glyphosate over non-target organisms. However, the accumulation of scientific evidence on the adverse effects of glyphosate and degradation metabolites (AMPA), over different organisms, including soil microorganisms, plants, insects, fishes, among other animals, humans included, highlighted the impact of the extensive use of glyphosate worldwide. These facts increase the relevance of the development of alternatives for its elimination from different polluted environments, as physicochemical and biological approaches. Bioremediation is a suitable alternative for the treatment of glyphosate-related pollution. Several microorganisms, bacteria, and fungi have great potential for the biodegradation of this herbicide. The present review condensed a recent reports of the use of microorganisms as alternatives for glyphosate degradation, the most important bacterial genera reported being *Achromobacter*, *Bacillus*, *Burkholderia*, *Ochrobactrum*, and others, while the genera *Aspergillus* and *Trichoderma* were the most important among fungi. Due to the inclusion of glyphosate as a probable carcinogen by the IARC, bioremediation studies of this herbicide will become more relevant in the next years. In summary, additional studies are needed to evaluate the presence and levels of glyphosate and its related metabolites in the environment. In the present review, several studies related to the evaluation of microorganisms for glyphosate biodegradation were identified. However, studies of the application of microorganisms in bioremediation strategies are limited. In situ studies on the application of these microorganisms in glyphosate remediation approaches are urgently needed. Fungi and algae are emerging groups of microorganisms reported in glyphosate biodegradation studies. There is a need to evaluate a higher number of fungal and algae species to establish the real potential of such microorganisms in glyphosate biodegradation. As future trends, studies applying the OMICs technologies could help to understand in a better way the mechanisms employed by such microorganisms for glyphosate degradation and the strategies employed to avoid its adverse metabolic effects.

## Figures and Tables

**Table 1:** Recent reports in microbial glyphosate degradation.

Species	Concentration (mg/L)	Degradation (%)	System	Degradation Pathway ^§^	Reference
**Bacteria**					
*Achromobacter denitrificans* SOS5	253	56	In vitro	glyphosate oxidoreductase	[152]
*Achromobacter insolitus* SOR2		47		glyphosate oxidoreductase	
*Achromobacter xylosoxidans* SOS3		37		glyphosate oxidoreductase	
*Agrobacterium tumefaciens* CHLDO		40		glyphosate oxidoreductase and C–P lyase	
*Ochrobactrum haematophilum* SR		41		glyphosate oxidoreductase	
*Bacillus megaterium*	5–25	70–71	In vitro	NR	[153]
*Acidovorax* sp. CNI26	22.31	NR	In vitro	C–P lyase	[139]
*Agrobacterium tumefaciens* CNI28				glyphosate oxidoreductase and C–P lyase	
*Ensifer* sp. CNI115				glyphosate oxidoreductase	
*Novosphingobium* sp. CNI35				glyphosate oxidoreductase and C–P lyase	
*Ochrobactrum pituitosum* CNI52				glyphosate oxidoreductase and C–P lyase	
*Bacillus aryabhattai* FACU	50, 100, 150, 200 and 250	NR	In vitro	glyphosate oxidoreductase	[154]
*Comamonas odontotermitis* P2	500	90	In vitro	glyphosate oxidoreductase and C–P lyase	[155]
*Ochrobactrum* sp.	50	60	In vitro	NR	[156]
*Pseudomonas citronellolis*					
*Bacillus cereus*	169	38	In vitro	C–P lyase	[157]
*Lysinibacillus sphaericus*	679 g/Kg *	79	In situ	C–P lyase	[158]
*Bacillus subtilis*	250	90	In vitro	glyphosate oxidoreductase and C–P lyase	[159]
*Rhizobium leguminosarum*		88			
*Streptomyces* sp.		89			
*Bacillus cereus*	3, 100, 7200 and 14,400	86, 73 and 57	In situ	glyphosate oxidoreductase and C–P lyase	[160]
*Pseudomonas aeruginosa*		76, 85 and 47			
*Ochrobactrum intermedium* Sq20	500	100	In vitro	C–P lyase	[145]
*Burkholderia* sp. AQ5-13	50	91	In vitro	NR	[144]
*Burkholderia vietnamiensis* AQ5-12		74			
*Ensifer* sp. AC01b	5072	44	In vitro	NR	[161]
*Rhizobium* sp. SCAUS14		41			
*Sinorhizobium saheli* OP3-1		39			
*Achromobacter* sp. MPK 7A	500	60	In vitro	C–P lyase	[162]
**Fungi**					
*Aspergillus* 2B112	500	60	In vitro	NR	[163]
*Penicillium* 4A21		26		glyphosate oxidoreductase and C–P lyase	
*Trichoderma*		8		NR	
*Aspergillus flavus* EFB01	100	19.9	In vitro	glyphosate oxidoreductase	[164]
*Aspergillus flavus* JN-YG-3-5		85.6			
*Aspergillus fumigatus* FJAT-31052		84.7			
*Aspergillus niger* APBSDSF96		84.8			
*Penicillium simplicissimum* SNB-VECD11G		84.7			
*Trichoderma gamsii* P2-18		84.2			
*Trichoderma harzianum* MT871998	200, 400, 600, 800 and 1000	78.1	In vitro	NR	[165]
*Aspergillus oryzae* AM1	1690	57	In vitro	glyphosate oxidoreductase	[166]
*Aspergillus oryzae* A-F02	500	66.9	In vitro	glyphosate oxidoreductase and C–P lyase	[142]
**Algae**					
*Oscillatoria limnetica*	5	85	In vitro	NR	[167]
	10	38			
	15	27			
	20	75			

^§^ Microorganisms with the same reference share the same glyphosate biodegradation pathway unless otherwise listed. * Concentration expressed in grams of herbicide by kilogram of soil. NR: not reported.

## Data Availability

Not applicable.
